# Genome sequencing and transcriptome analysis of *Trichoderma reesei* QM9978 strain reveals a distal chromosome translocation to be responsible for loss of *vib1* expression and loss of cellulase induction

**DOI:** 10.1186/s13068-017-0897-7

**Published:** 2017-09-07

**Authors:** Christa Ivanova, Jonas Ramoni, Thiziri Aouam, Alexa Frischmann, Bernhard Seiboth, Scott E. Baker, Stéphane Le Crom, Sophie Lemoine, Antoine Margeot, Frédérique Bidard

**Affiliations:** 1 IFP Energies Nouvelles, 1-4 Avenue de Bois-Préau, 92852 Rueil-Malmaison, France; 2 Molecular Biotechnology, Research Division Biochemical Technology, Institute of Chemical Engineering, TU-Wien, 1060 Vienna, Austria; 3 Earth and Biological Sciences Directorate, Pacific Northwest National Laboratory, Richland, WA 99354 USA; 4 Evolution Paris Seine-Institut de Biologie Paris Seine (EPS-IBPS), Sorbonne Universités, UPMC Univ Paris 06, Univ Antilles, Univ Nice Sophia Antipolis, CNRS, 75005 Paris, France; 5 École normale supérieure, PSL Research University, CNRS, Inserm, Institut de Biologie de l’École normale supérieure (IBENS), Plateforme Génomique, 75005 Paris, France; 6 Present Address: Genetics of Biofilms Unit, Department of Microbiology, Institut Pasteur, 25-28 Rue du Dr Roux, 75015 Paris, France

**Keywords:** *Trichoderma reesei*, Genome analysis, Transcriptome, *vib1*, Cellulase production, Promoter, Translocation

## Abstract

**Background:**

The hydrolysis of biomass to simple sugars used for the production of biofuels in biorefineries requires the action of cellulolytic enzyme mixtures. During the last 50 years, the ascomycete *Trichoderma reesei*, the main source of industrial cellulase and hemicellulase cocktails, has been subjected to several rounds of classical mutagenesis with the aim to obtain higher production levels. During these random genetic events, strains unable to produce cellulases were generated. Here, whole genome sequencing and transcriptomic analyses of the cellulase-negative strain QM9978 were used for the identification of mutations underlying this cellulase-negative phenotype.

**Results:**

Sequence comparison of the cellulase-negative strain QM9978 to the reference strain QM6a identified a total of 43 mutations, of which 33 were located either close to or in coding regions. From those, we identified 23 single-nucleotide variants, nine InDels, and one translocation. The translocation occurred between chromosomes V and VII, is located upstream of the putative transcription factor *vib1*, and abolishes its expression in QM9978 as detected during the transcriptomic analyses. Ectopic expression of *vib1* under the control of its native promoter as well as overexpression of *vib1* under the control of a strong constitutive promoter restored cellulase expression in QM9978, thus confirming that the translocation event is the reason for the cellulase-negative phenotype. Gene deletion of *vib1* in the moderate producer strain QM9414 and in the high producer strain Rut-C30 reduced cellulase expression in both cases. Overexpression of *vib1* in QM9414 and Rut-C30 had no effect on cellulase production, most likely because *vib1* is already expressed at an optimal level under normal conditions.

**Conclusion:**

We were able to establish a link between a chromosomal translocation in QM9978 and the cellulase-negative phenotype of the strain. We identified the transcription factor *vib1* as a key regulator of cellulases in *T. reesei* whose expression is absent in QM9978. We propose that in *T. reesei*, as in *Neurospora crassa*, *vib1* is involved in cellulase induction, although the exact mechanism remains to be elucidated. The data presented here show an example of a combined genome sequencing and transcriptomic approach to explain a specific trait, in this case the QM9978 cellulase-negative phenotype, and how it helps to better understand the mechanisms during cellulase gene regulation. When focusing on mutations on the single base-pair level, changes on the chromosome level can be easily overlooked and through this work we provide an example that stresses the importance of the big picture of the genomic landscape during analysis of sequencing data.

**Electronic supplementary material:**

The online version of this article (doi:10.1186/s13068-017-0897-7) contains supplementary material, which is available to authorized users.

## Background

In order to bypass the current dependence on fossil resources and to reduce carbon dioxide emissions, cellulosic and hemicellulosic polymers are considered as environmentally clean and renewable energy sources and are used for the production of bioethanol and platform chemicals. Lignocellulosic material as found in agricultural crop residues, grasses, and wood must undergo physical and chemical pre-treatment before fermentable sugars can be released by the action of cellulosic enzymes. The production costs of those enzyme cocktails are still very high and present a limiting factor in the production of second-generation biofuels [1].

*Trichoderma reesei*, a filamentous fungus naturally found growing on decaying wood, is well known for its high capacity to secrete large amounts of cellulolytic enzymes and some strains are commonly used as industrial protein production hosts [2, 3]. All *T. reesei* mutants which are currently used for industrial enzyme production or academic research originate from a single wild-type strain (QM6a) isolated on the Solomon Islands during the second world war due to its capability to degrade the cotton fabric of tents of the US army troops stationed there [4]. The strain was part of the US Army Quartermaster (QM) culture collection, and its cellulase system has been characterized over several decades [5]. During the first oil crisis in the 1970s, improved *T. reesei* strains were chosen for industrial enzyme production [5–7].

**Fig. 1:**
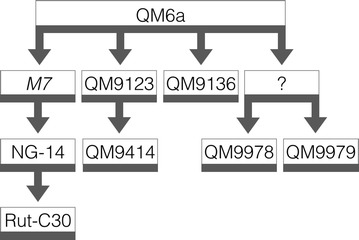
Pedigree of the *T. reesei* strain QM9978 and its relationship to the wild-type isolate QM6a. The progeny of the original isolate QM6a was derived by classical mutagenesis using UV light, irradiation by linear particle accelerators, and/or *N*-methyl-*N′*-nitro-*N*-nitrosoguanidine (NTG). Strain M7 is indicated in* italics*, as it is no longer available. Strains QM9136, QM9978, and QM9979 are deficient in cellulase production. Sequence analysis identified QM9978 to originate from a strain that is unknown. It is therefore pictured with a question mark

Since then, different mutagenesis programs have produced several strain lineages of *T. reesei*, some with higher enzyme production capabilities and some with lower or absent cellulase production [8] (Fig. 1). Most notably, the strain lineage developed at the Rutgers University in the 1970s produced strain Rut-C30, with the highest cellulase production levels available for academic research [7]. Strains QM9414 and QM9978 are both a product of mutagenesis programs at the US Army Natick laboratories. Whereas QM9414 serves as a common reference strain due to its moderately improved cellulase production, QM9978, QM9979, and QM9136 have cellulase-negative phenotypes providing an opportunity for understanding the mechanisms underlying cellulase regulation [9]. Indeed, the genetic causes leading to cellulase overproduction remain relatively uncharacterized. Only a few examples exist where a specific genetic change could be linked to an improved or decreased cellulase production [8, 10–16]. Whole genome sequence analysis of several strains has contributed to the understanding of the involved mechanisms [13, 14, 16–18] (Fig. 1).

In this work, we chose the strain QM9978 as a model to understand cellulase production as it is a cellulase-negative strain and the cause for it was yet unknown.

**Fig. 2:**
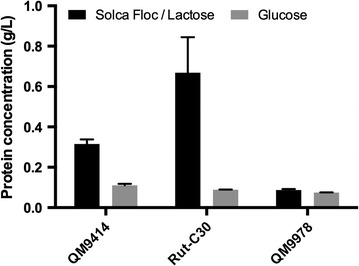
Extracellular protein production in the reference strains QM9414, Rut-C30, and QM9978. Protein concentration in the supernatant was determined from cultures on liquid minimal medium supplemented with 10 g/L Solka-floc cellulose/lactose or glucose. Supernatant was harvested after 3 days and analyzed by the Lowry method using BSA as a standard. Data of two biological and three technical replicates are shown

Earlier studies on strain QM9978 suggested that there is a defect in the cellulase induction process but not in the basal expression of cellulase genes [9]. The uptake of cellobiose and sophorose is not affected suggesting that its cellulase-negative phenotype is most likely a result of a defective component specifically involved in the regulation of cellulase expression rather than during induction [19]. Another study using gene expression analysis of QM9414 and QM9978 on sophorose performed by the rapid subtraction hybridization (RaSH) method led to the identification of 18 differentially expressed genes, among which was the homologue of the *N. crassa* clock modulator *vivid*, the translation elongation factor 1α, and the transcriptional activator Hap1 [20]. Phenotypically, QM9978 forms less conidia than QM6a when grown on potato dextrose agar (PDA), fails to produce sufficient cellulases on any tested medium, and shows no growth when cultivated in liquid medium with cellulose as the sole carbon source [9, 20, 21] (Fig. 2).

In an effort to identify new components of the cellulase gene regulation network of *T. reesei*, we used a whole genome sequencing and transcriptomic analysis approach comparing the cellulase-negative strain QM9978 to the wild-type strain QM6a. We show that—among a few other mutations that were unlikely to have a significant impact on cellulase formation—this strain contains a chromosomal translocation upstream of the promoter region of the *N. crassa* homologue *vib*-*1* (vegetative incompatibility blocked) and that *vib1* expression in QM9978 is absent. The cellulase production in this strain can be restored by introduction of an additional *vib1* copy and by *vib1* overexpression. We further investigated the role of *vib1* in the improved cellulase producer strains QM9414 and Rut-C30 and show that cellulase gene regulation in the three strains follows different regulatory pathways.

## Results

### Genome analysis of QM9978

To identify the genetic differences between QM6a and QM9978 leading to the cellulase-negative phenotype, we performed whole-genome high-throughput Illumina sequencing of QM9978 (for details, see “Methods” section). The comparative sequence analysis identified a total of 43 mutations in QM9978, of which 33 were found in the coding region of genes or in the assumed promoter (800 bp upstream) or terminator (200 bp downstream) region. Of those, 23 were characterized as SNVs (single-nucleotide variants), eight as InDels (small insertions and deletions), and two as deletions with 7 and 47 bp missing, respectively (Table 1). They were located on 22 different scaffolds. The number of mutations in QM9978 is about three times higher than in QM9136, another QM6a derivative cellulase-negative strain used for the study of the cellulolytic system of *T. reesei* [18] (Fig. 1). Whole genome sequence analysis of QM9136 identified a truncation in the major cellulase regulator *xyr1* to be responsible for the cellulase-negative phenotype of this strain [18]. The gene encoding *xyr1* in QM9978 is intact, and therefore another cause must exist for its disability to degrade cellulose.

**Table 1:** Mutations found in *T. reesei* QM9978 compared to WT QM6a

SNP_id	Mutation	Event	Position	Transcript	Element	Annotation
1_2509832	SNV	A > G		Trire2:119839	Intron	Putative cyanamide hydratase
1_753158	SNV	T > G		Trire2:21170	Intron	Putative 40S ribosomal protein S12
1_753159	SNV	C > T		Trire2:119626	Prom	Putative 40S ribosomal protein S22
1_96633	Transloc	t(1;16)(96,633;631,551)		Trire2:54675	Upstream	Putative transcription factor VIB1
10_21554	InDel	−1:G	+1671	Trire2:121962	Exon	Blue-light regulator 1 BRL1
11_325040	SNV	G > C	+583	Trire2:4231	Exon	Putative siderophore regulation protein
12_146369	InDel	−1:T		Trire2:108419	Prom	Putative protein of unknown function
12_146369	InDel	−1:T		Trire2:122376	Prom	Putative protein of unknown function
12_778518	SNV	T > C		Trire2:108592	Intron	Putative leucine aminopeptidase
12_842068	SNV	G > A	+776	Trire2:79153	Exon	Putative 5-oxoprolinase OXP1
13_429746	SNV	G > A		Trire2:79300	Intron	Putative sphingolipid long chain base-responsive protein
13_839848	SNV	T > C		No		
14_189528	InDel	1:A		Trire2:79485	Term	Putative peptide *N*-glycanase degradation
15_427120	SNV	C > G		Trire2:4767	Prom	Putative biotin synthase
16_631551	Transloc	t(1;16)(96,633;631,551)		Trire2:80028	Upstream	Putative ABC transporter
17_646809	SNV	G > T		Trire2:65965	Prom	Putative S-adenosyl-l-methionine-dependent methyltransferase
20_145726	SNV	T > C	+293	Trire2:55671	Exon	Putative NAD(P)-binding Rossmann-fold domain protein
2_618372	SNV	C > T	+2098	Trire2:66913	Exon	Putative phosphatidylinositol 3-kinase
24_496758	InDel	−1:G		Trire2:123658	Intron	Putative guanylate-binding domain protein
26_169591	SNV	CG > AC	+2901	Trire2:51893	Exon	Putative lipid body protein ppoC
26_331506	SNV	G > A		Trire2:68803	Term	Putative RTA-1 domain protein
28_280867	InDel	−1:T		No		
29_224118	SNV	T > C		No		
3_1061204	InDel	−7:ATATCAT		No		
3_1353739	SNV	G > A	+635	Trire2:104458	Exon	Putative protein of unknown function
31_146257	SNV	A > G	+427	Trire2:69881	Exon	Putative pyoverdine/dityrosine biosynthesis protein
33_52840	InDel	−1:T		No		
37_85324	SNV	G > A		Trire2:124181	Intron	Putative DML1 protein
4_1236402	SNV	A > T		No		
4_1680892	SNV	G > A	+127	Trire2:41761	Exon	Putative vacuolar iron transporter
4_890445	SNV	A > T		Trire2:75921	Prom	Putative tRNA-dihydrouridine synthase 1
4_890446	SNV	A > T		Trire2:75921	Prom	Putative tRNA-dihydrouridine synthase 1
43_42477	SNV	G > A	+1144	Trire2:6057	Exon	Putative ubiquinol cytochrome-c reductase assembly protein
6_289426	SNV	G > T		No		
6_289431	SNV	C > G		No		
6_622255	SNV	G > C		Trire2:105968	Prom	Putative PcbC family protein
8_1174800	InDel	−7:GGAGGTC		Trire2:39911	Intron	Putative S-adenosylmethionine-homocysteine methyltransferase
8_1290905	InDel	−1:A	+452	Trire2:47897	Exon	Putative ABC transporter
9_1123734	SNV	G > T		No		
9_223955	InDel	−1:A		Trire2:77836	Prom	Putative protein of unknown function
9_475049	InDel	−1:G		No		

The SNP_id number gives the position on the scaffold as obtained by Illumina sequencing. The presented transcript ID corresponds to the protein ID in the JGI database (http://genome.jgi.doe.gov)

*Trichoderma reesei* QM9123 was, until now, commonly believed to be the parental strain of QM9978. Since the genome sequence of QM9123 was published in 2010, we performed a comparison to QM9978 and showed that none of the 20 mutations identified in QM9123 is shared by QM9978 [16]. We therefore exclude the possibility that QM9123 is the parental strain of QM9978 and present an up-dated version of the *T. reesei* strain lineage in Fig. 1. No detailed record on the generation of the strain QM9978 and the closely related strain QM9979, which is also cellulase negative, exists, but we assume that the high number of mutations is a result of two or more subsequent mutagenesis events. Sequence comparison of QM9978 to QM9979 showed common mutations between those two strains indicating that they originate from the same parental strain (F.B., personal communication). Compared to high cellulase producer strains like *T. reesei* NG14 (one InDel, 78 SNVs, and nine large structural variations) and Rut-C30 (one deletion, two InDels, and 106 SNVs additional to NG14), the number of mutations is comparably low, probably because of alternative mutagenesis methods, and number and severity of treatments [14, 16, 17, 22].

From the 19 genes where a mutation occurred in the coding region, only the role of the blue-light receptor BLR1 in cellulase regulation has been studied in detail. Although BLR1 and BLR2 are the key regulators of the blue-light response in fungi and conidiation, it was shown that *blr1* is not essential for cellulase expression [23] (Table 1).

**Fig. 3:**
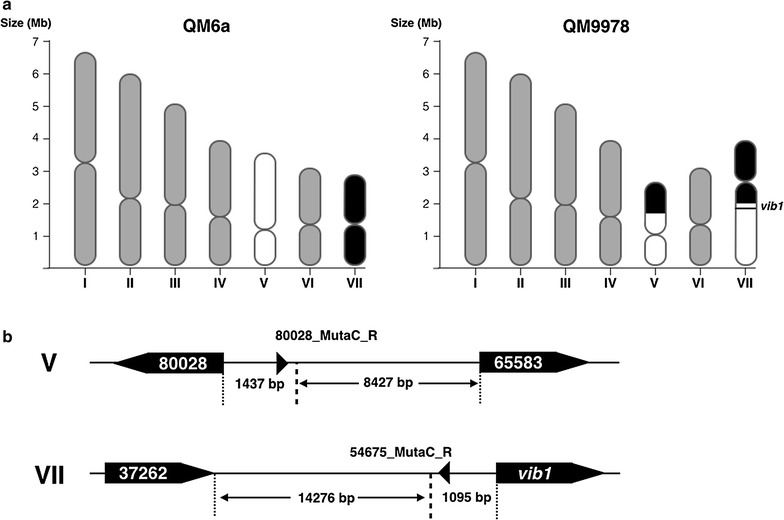
Analysis of the chromosomal translocation in *T. reesei* QM9978. **a** Schematic depiction of the seven chromosomes in *T. reesei* QM6a and QM9978 showing the translocation. **b** Verification of the translocation by PCR was performed with oligonucleotides 800_MutaC_R and 54675_MutaC_R binding outside of the DNA break. The resulting DNA-fragment was analyzed by Sanger sequencing in order to identify the exact translocation point

Our research focused on the identified deletions and those mutations were subsequently verified by PCR analysis using primers flanking the deleted fragment. For two predicted deletions (on scaffold 1 and scaffold 16), we could not confirm the results obtained by Illumina sequencing as no PCR product was obtained. The recent genome assembly of *T. reesei* using chromosomal contact data in a computational approach identified scaffolds 1 and 16 to be located on chromosomes number five and seven, respectively [24–26]. Upon close examination of our sequencing data using the Integrative genome viewer program (http://software.broadinstitute.org/software/igv/) combined with the information on the Joint Genome Institute homepage (http://genome.jgi.doe.gov), it appeared that what we originally believed to be a deletion event could in reality be a translocation resulting from a chromosome break 1095 bp upstream of the ATG start codon of Trire2:54675 located on chromosome VII (Fig. 3a). To verify this hypothesis, we designed oligonucleotides flanking the assumed DNA break. Only PCRs with the primer combination 80028_MutaC_R and 54675_MutaC_R binding on the two different chromosomes resulted in an amplicon (Fig. 3b). Sequencing of this amplicon identified the region where the DNA breaks occurred. As a result from the translocation, the gene Trire2:37262 (a putative ubiquitin-protein ligase) upstream from Trire2:54675 on chromosome VII is replaced by Trire2:80028, a putative ABC transporter on chromosome V (Fig. 3b).

### Gene expression profiling in *T. reesei* QM9978

Changes in gene expression between QM9978 and QM6a were analyzed in order to define the impact of mutations at the transcriptional level. Strains were grown on d-glucose for approximately 24 h until the carbon source was depleted from the medium (less than 3 g/L glucose remaining). Then, a lactose pulse of 10 g/L was added to the medium as lactose is known to be a strong inducer of cellulases [27]. Total RNA of two biological replicates was extracted at three different time points: 0 h (after carbon source depletion and before lactose addition), 24 and 48 h after the lactose pulse. Statistical treatments and differential analyses were performed using DESeq 1.8.3. A threshold of two for the log2 ratios with an adjusted *p* value <0.001 was used to identify genes differentially expressed (DE). Gene functions were identified by the aid of a manually annotated *T. reesei* genome database proprietary to IFPEN.

Comparison of the gene expression in QM9978 to QM6a identified a total of 785 genes differentially expressed (DE) in at least one time point. The number of down-regulated genes slightly increases during the time course, whereas the number of up-regulated genes remains stable. Interestingly, DE genes are mainly down-regulated, meaning their expression is lower in QM9978 then in QM6a. We compared the gene expression data with our sequencing results and identified five DE genes, which were affected by mutations. The genes were characterized by homology search using the NCBI BLASTp tool (https://blast.ncbi.nlm.nih.gov/Blast.cgi) and the Joint Genome Institute genomics resource database (http://genome.jgi.doe.gov). The annotation was additionally verified manually using the FUNGIpath database (http://embg.igmors.u-psud.fr/fungipath/). This way we identified ID51893—an orthologue of the lipid body protein ppoC (*A. nidulans*) involved in coordinating sexual and asexual sporulation [28], ID54675—a homologue of the transcription factor *vib*-*1* involved in vegetative incompatibility and cellulose utilization in *N. crassa* [29], ID68803—a predicted RTA1-domain protein, and ID108418 and ID122376—two hypothetical proteins of unknown function.

From the total of 785 genes, only 153 genes (23%) were DE at all time points. In this gene set, we find 14 glycoside hydrolases (including *cbh2*, *xyn2*, and *agl1*), eight putative transporters (including *crt1*), and four transcription factors including *vib1* (ID54675) and *azf1/sah*-*1* (ID61055) as identified by homology search as described above [30]. The *N. crassa* homologues of these two transcription factors have been shown to be involved in glucose signaling and to be up-regulated during growth on the silvergrass *Miscanthus* [31]. Of these, 153 genes two are impacted by mutation: *vib1* and the putative RTA1-domain protein (ID68803).

Among the four genes surrounding the translocation on chromosomes V and VII identified during our sequence analysis, only *vib1* and Trire2:80028, a putative ABC transporter whose expression is diminished in QM9978, show altered expression levels (Fig. 3).

Blastp analysis showed that *T. reesei vib1* shows 49% similarity to *N. crassa vib*-*1* [29, 32]. Since *N. crassa vib*-*1* has been shown to be essential for cellulase production in *N. crassa*, we investigated whether a link could be established between the translocation, *vib1* expression, and the loss of cellulase production in the QM9978 strain.

### Analysis of the role of *vib1* in cellulase induction

#### Constitutive expression of vib1 restores cellulose utilization in QM9978

**Fig. 4:**
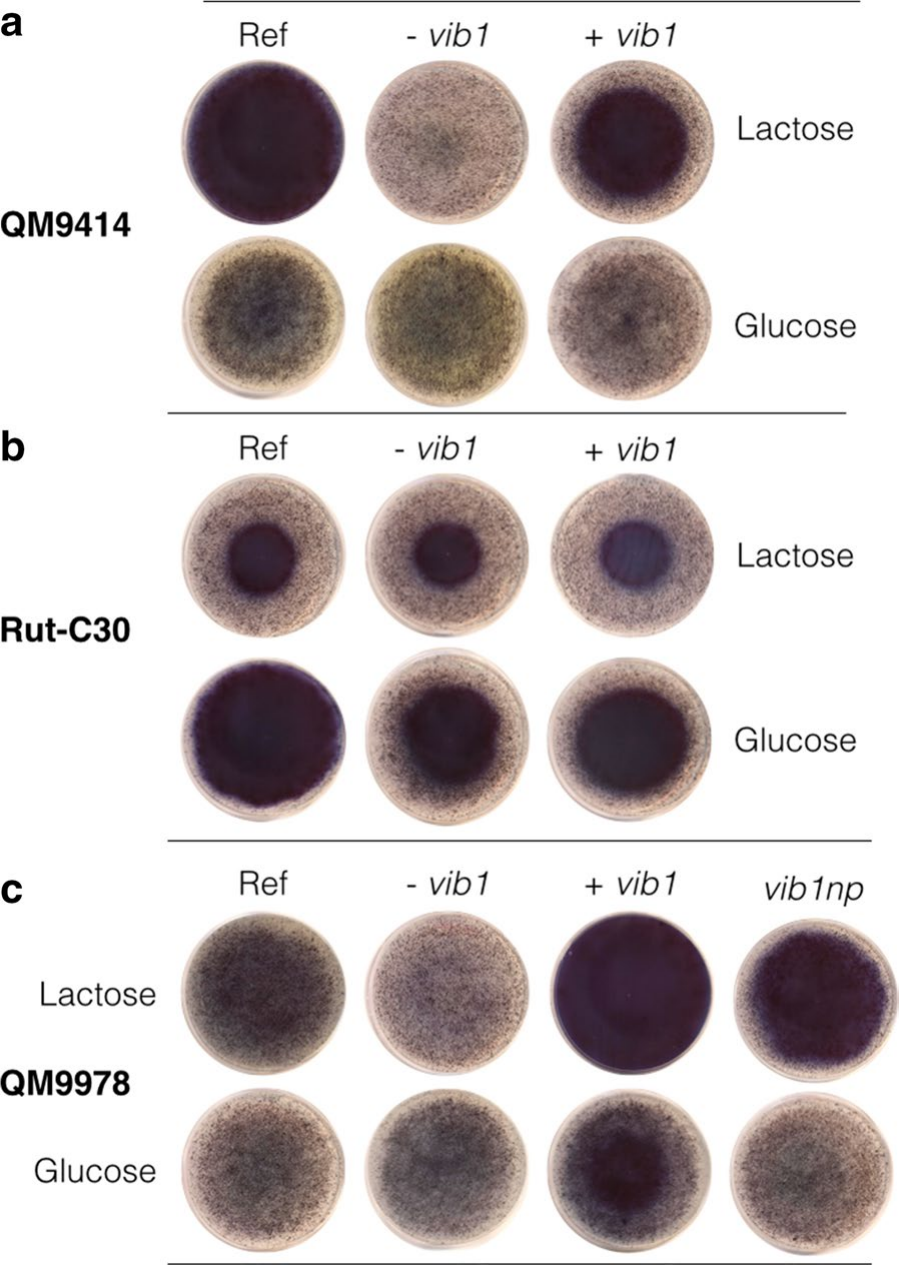
Detection of cellulase production by *vib1* mutants on AZCL-HE-Cellulose. Comparison of *vib1* deletion (−*vib1*) and overexpression *(*+*vib1)* strains of **a** QM9414, **b** Rut-C30, and **c** QM9978 on minimal medium supplemented with glucose or lactose (1% w/v) and AZCL-HE-Cellulose (0,1% w/v). *Blue* halos on the agar plates result from degradation of the AZCL-HE-Cellulose substrate releasing a *blue dye* into the agar. Pictures were taken from the *bottom* side of the plates. The high enzyme production of Rut-C30 on glucose is due to the bigger colony size of this strain on glucose. For a comparison of colony size to enzyme production see Additional file 1: Figure S1, depicting the same plates from above. Pictures were taken after 4 days of incubation. For each strain, two biological replicates (individual clones) were assayed and one is shown representatively

To determine whether *vib1* is involved in cellulose deconstruction by *T. reesei*, we constructed a *vib1* overexpression QM9978 strain (QM9978 +*vib1*) by ectopic integration of the gene under the control of the strong constitutive promoter of *T. reesei* glyceraldehyde-3-phosphate dehydrogenase (*gpd*) [33]. For two independent clones, we monitored enzyme production during 4 days using a fast screening assay based on azurine-crosslinked cellulose (AZCL-HE-cellulose) that releases a soluble blue dye into the agar medium after enzymatic hydrolysis by endoglucanases, in which the size of the blue halo on an agar plate corresponds to the amount of secreted cellulases. Plates supplemented with 10 g/L glucose or lactose (for cellulase induction) and 1 g/L AZCL-HE-cellulose were used and the results of one clone per strain are representatively shown in Fig. 4 and Additional file 1: Figure S1. Strain QM9978 failed to secrete a sufficient amount of cellulases on lactose to decompose the AZCL-HE polymer, whereas the agar of the QM9978 +*vib1* strain was stained blue due to enzymatic hydrolysis. To verify that no residual VIB1 activity remains in QM9978, we constructed the *vib1* deletion strain QM9978 −*vib1*. Like QM9978, this strain was unable to decompose AZCL-HE-cellulose, indicating that the mutation in QM9978 equals a null phenotype (Fig. 3).

**Fig. 5:**
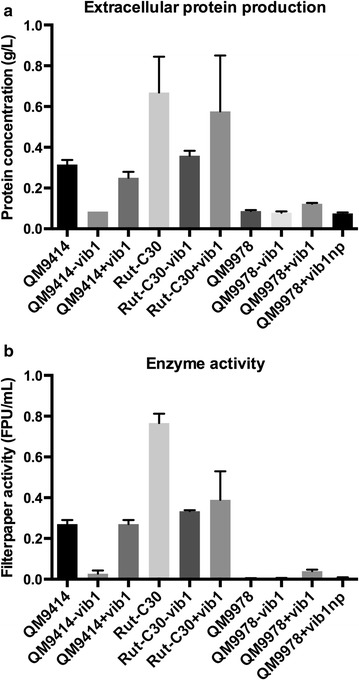
Comparison of extracellular protein production and cellulase activity in *vib1* deletion and overexpression strains. **a** Protein concentration was determined by the Lowry method and is shown for *−/+vib1* strains and their respective reference strains QM9414, Rut-C30, and QM9978 using a BSA standard. **b** Filter paper activity was determined for the same samples, allowing a direct comparison between protein content and enzyme activity in the supernatant. The samples were prepared from liquid minimal medium supplemented with 10 g/L Solka-floc cellulose/lactose as carbon source. Supernatant was harvested after 3 days. Data of two biological and three technical replicates are shown

Since the data from the plate assay suggested a strong impact of *vib1* on cellulase production, we produced liquid cultures grown on 10 g/L lactose/Solka-floc (cellulase induction) and on 10 g/L glucose (repression) to determine the protein content in the supernatant and the volumetric enzyme activity (see “Methods” for details on the protocol). Both the extracellular protein concentration and the filter paper activity were found to be significantly increased in strain QM9978 +*vib1* compared to QM9978 (*p* < 0.0001 and *p* < 0.01, respectively) (Fig. 5).

**Fig. 6:**
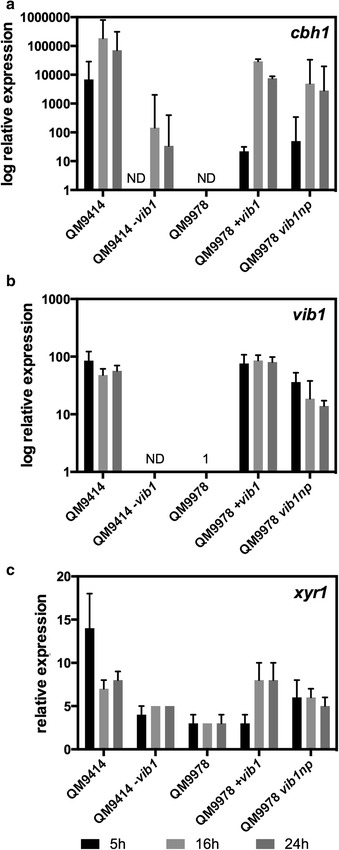
VIB1 is required for cellulase production and signaling. Expression levels for the cellulase gene *cbh1* **a**, the transcription factor *vib1* **b**, and the main positive regulator *xyr1* **c** were assessed in *−/+vib1* strains versus WT after a shift from glycerol to 1% (v/w) lactose. Gene expression levels were measured by relative quantitative PCR using *tef1* and *sar1* as a control and normalized to expression level at time point zero (24 h growth on glycerol) for *cbh1* and *xyr1* or to expression level in QM9978 for *vib1*. Samples were taken 5 h (*black*), 16 h (*light gray*), and 24 h (*dark gray*) after replacement. Mean values ± standard error of triplicate data from two biological replicates are shown. *ND* not detected

The translocation in QM9978 occurred 1095 bp upstream of the *vib1* ATG start codon. Therefore, the indicated promoter of approximately 1 kb remains unaffected [34, 35]. To test whether the abolished *vib1* expression in QM9978 is due to the missing 5′ upstream region, we constructed a QM9978 strain expressing *vib1* under the control of 850 bp of its native promoter, ectopically integrated. This allowed us to investigate the role of the 5′ region absent in QM9978, for example, if activator-binding sites were affected. The resulting strain QM9978_*vib1np* had an enzyme secretion capacity comparable to QM9978 +*vib1* on plate test using AZCL-HE-cellulose (Fig. 4). These results were confirmed by real-time qPCR analysis, where we detected a significant accumulation of *cbh1* (*cel7a*) transcript, both for the overexpression strain QM9978 +*vib1* and for QM9978_*vib1np* (Fig. 6a). Expression levels in both strains remained lower compared to strain QM9414, probably due to the fact that strain QM9414 has undergone a different mutagenesis treatment during strain engineering leading to higher expression of *chb1* than a cellulase-restored QM9978 strain.

Analysis of *vib1* transcription in QM9978 +*vib1* and QM9978_*vib1np* confirmed that *vib1* transcripts were present at levels equivalent to QM9414, a strain with unimpaired *vib1* expression (Fig. 6b).

#### vib1 deletion, but not overexpression, has an effect on cellulase induction in higher producer strains

Strain QM9414 has been extensively used as a reference strain in the literature when comparing different mutations due to its capacity to produce a measurable amount of cellulases (QM6a producing cellulase levels below the detection limit of most assays). In addition to QM9414, we chose strain Rut-C30 as this is the highest enzyme producer strain (reported to produce 30 g/L enzymes, depending on culture conditions) available for public research and is also used in industrial applications [36]. We performed our study in Rut-C30 to show if an industrial strain originating from the Rut lineage can be further improved by *vib1* overexpression.

To gain a better understanding of the role that *vib1* plays in cellulase induction we made use of the different genetic backgrounds of strain QM9414 and Rut-C30 and constructed the strains QM9414 ±*vib1* and Rut-C30 ±*vib1* by deleting *vib1* or ectopically introducing an additional gene copy under the control of the strong constitutive *gpd1* promoter, equivalent to the approach used in QM9978.

Screening on AZCL-HE-Cellulose revealed that *vib1* deletion had a negative effect on cellulase production in QM9414 and Rut-C30, whereas no effects could be observed for the overexpression (Fig. 4). This finding was further confirmed by the measurement of extracellular protein concentration and enzyme activity in liquid culture (minimal media supplemented with Solka-floc and lactose) and reflected in the reduced expression of *cbh1* (*cel7a*) in the *Δvib1* strains (Figs. 5, 6). We therefore conclude that overexpression of *vib1* does not additionally improve cellulase production in those two strains.

Since previous studies have identified XYR1 as a major regulator of cellulase gene expression and *xyr1* deficiency to be linked to a cellulase-negative phenotype [37], we determined *xyr1* transcript levels in the QM9978 and QM9414 strains. Deletion of *vib1* in QM9414 significantly reduced *xyr1* expression, whereas over-/expression of *vib1* increased *xyr1* levels in QM9978 (*p* < 0.001) (Fig. 6c). This finding indicates that XYR1 could function downstream of VIB1 and the reduced *xyr1* expression could contribute to the cellulase-negative phenotype of −*vib1 T. reesei* strains.

## Discussion

The manipulation of *T. reesei* strains by random mutagenesis and subsequent selection procedures has led to the isolation of strains with cellulase production capacities surpassing 100 g/L in industrial culture conditions [38]. The exact identification of the genetic changes that are responsible for the cellulase overproduction relied for a long time on laborious complementation studies, which were not very successful. Advances in high-throughput sequencing and genomic profiling have significantly contributed to a lower cost of these technologies and thus offer a feasible alternative for the identification of mutations [14, 16–18]. Strains obtained by random mutagenesis often contain a multitude of mutations, and genome analysis of QM9978 resulted in the identification of 33 mutations found in the coding region of genes or their promoters, plus four genes found in the region of a chromosomal rearrangement. In order to try and reduce the number of candidate genes responsible for the cellulase-negative phenotype, we combined the genome sequence analysis with a transcriptomic study in cellulase inducing conditions. This tandem approach and clues from existing literature about the role of *N. crassa vib*-*1* in cellulase regulation led us to select *vib1* as the main candidate, despite the fact that the coding region and the close-by intergenic regions of the gene were not affected by the mutagenesis [39]. A sequencing approach alone probably would not have resulted in *vib1* being selected as prime candidate as the identified mutation is far away from the start codon. Considering distal genomic events and combining genome sequencing with a transcriptome analysis seems a robust approach for microbial strain “reverse-engineering.”

Indeed, analysis of the genomic landscape led to the identification of a translocation between chromosomes V and VII. It has been shown that DNA damage such as double-strand breaks can lead to incompletely cleared sites of repair causing gene silencing through epigenetic events [40, 41]. Histone modifications such as methylation, acetylation, or displacement are a common mechanism of gene regulation in filamentous fungi [42]. We speculated that the chromosome break led to histone modifications no longer favoring active transcription, since the translocation took place only 1 kb upstream of the *vib1* start codon. *Vib1* and Trire2:80028, a putative ABC transporter whose expression is also diminished in QM9978 when compared to QM6a, show altered expression levels. The other two genes involved (Trire2:65583 and Trire2:37262) are located at longer distances and therefore may be too far away to see their expression impacted (8427 and 14,276 bp, respectively, as compared to 1095 and 1437 bp). Another explanation would involve the rearrangement of regulator binding sequences (e.g., inhibitors of transcription) in the close proximity of the affected genes, thus having an unfavorable effect on gene expression.

*Vib1* was initially identified in *N. crassa*, where it was shown to be required for vegetative incompatibility [29]. Since then, several studies have shown that *vib1* and its homologs in other organisms are involved in the regulation of a diverse spectrum of metabolic processes including programmed cell death, antifungal compound production, and the response to carbon starvation, among others [43–46]. During a recent screening of a transcription factor deletion library in *N. crassa*, mutants unable to grow on cellulosic biomass were identified. Among them, *vib*-*1* was shown to be essential for cellulose degradation. This finding led us to the assumption that *vib1* may be involved in cellulase regulation in *T. reesei*, and the observed down-regulation in the QM9978 strain could be due to the relatively distal chromosomal translocation identified upstream. To rule out the possibility that the discovered balanced translocation deleted activator-binding sites positioned further upstream and which are required for active transcription of *vib1*, we re-introduced *vib1* +850 bp as promoter sequence, which are not affected by the translocation. Since this approach could restore cellulase production, we conclude that despite a functional promoter and gene, the new genomic environment arising from the rearrangement is responsible for the loss of *vib1* expression, either by epigenetic modifications or through other mechanisms involving, for example, the binding of distal transcriptional repressors.

Overexpression of *vib1* in QM9978 restored enzyme secretion in this strain, and gene deletion in QM9414 abolished cellulase production, confirming that *vib1* is required for cellulose degradation. Gene deletion in the industrial strain Rut-C30 resulted in reduced although not completely abolished cellulase production, indicating that additional factors contribute to cellulase induction, especially since overexpression of *vib1* had no effect on cellulase production for QM9414 and Rut-C30. We therefore conclude that *vib1* is not suitable for the amelioration of hyper-producing strains, as they probably already accumulated other mutations masking the effect of *vib1* overexpression.

The exact role of the different transcription factors in *T. reesei* during cellulase induction is not fully understood yet. Although most of the cellulase genes are regulated in a coordinated way, their relative expression levels differ in higher producers [47]. Overexpression or constitutive activation of *xyr1* lead to a significant elevation/deregulation of xylanase transcription levels and had an impact on cellulase induction in other studies [48, 49].

Our transcriptional analysis showed differential regulation of the main positive regulator *xyr1* depending on *vib1* expression. We observed no significant changes in *xyr1* expression during cultivation on lactose, which is consistent with recent findings where *xyr1* induction does not require metabolism of d-galactose [50, 51]. The significantly reduced expression of *xyr1* in −*vib1* strains could contribute to the cellulase-negative phenotype of QM9978, as *xyr1* deletion has been shown to abolish cellulase induction on cellulose and sophorose and to impair the induction of hemicellulase genes involved in xylan and arabinan degradation in *T. reesei*, *N. crassa*, and *A. niger* [52–54]. Since *xyr1* expression in QM9978 and QM9414 −*vib1* is only reduced and not completely abolished, other factors besides VIB1 must be involved in its regulation and in cellulase gene expression.

A recent model for cellulase regulation in *N. crassa* proposes the involvement of several conserved transcription factors and shows that VIB-1 functions upstream of the inducing signal, repressing CRE1 and COL26 and thus inducing CLR-1 and CLR-2 [39, 55, 56] to promote cellulase production. The *T. reesei* homologue of CLR-2 was found to be significantly up-regulated during growth on lactose but a detailed analysis of its function is still missing [50, 57]. Overexpression of *clr2* in QM9414 has shown that *clr2* (gene 26163 in the respective study) had only a minor effect on cellulase production in *T. reesei*, and a homologue of *clr*-*1*, which is essential for cellulase production on *N. crassa*, is missing in *T. reesei* [13]. BglR, the COL26 homologue of *T. reesei*, was not regulated during growth on lactose in our study, neither in the QM6a wild type nor in QM9978. A recent study identified *col*-*26* to be involved in starch signaling in *N. crassa* but no data are available yet for *T. reesei* [58]. Our transcriptomic analysis showed no difference in *bglR*-expression in strains expressing/not expressing *vib1*. We therefore cannot conclude that *bglR* plays a major role in *vib1* downstream signaling in *T. reesei*. Whether a synergy between *cre1*, *bglR*, and *vib1* exists in *T. reesei*, like it does in *N. crassa,* remains for the moment unclear. Transcriptomic analysis performed previously by our lab on strains NG14 and RUT-C30 shows an enhanced expression of *vib1* in RUT-C30 during growth on lactose [59]. Since the major difference between NG14 and RUT-C30 is a partial deletion of *cre1* in RUT-C30, it may be speculated that in *T. reesei*, contrary to *N. crassa*, *vib1* acts downstream of the inducing signal, which would also explain why *bglR* is not differentially expressed in a *Δvib1* background.

In a study performed by Schmoll et al., 18 genes specifically expressed in QM9978 compared to QM9414 were identified [20]. From those genes, we find six which are differentially expressed in our study. The differences in experimental conditions and reference strains may explain why a different gene set was identified by Schmoll et al. compared to our analysis and why *vib1* was not detected as differentially expressed.

## Conclusions

Our analysis used genomic data in combination with gene expression analysis and revealed a chromosomal recombination event to be responsible for the cellulase-negative phenotype of QM9978. We identified VIB1 as a transcription factor required for the degradation of lignocellulosic biomass whose expression is absent in QM9978. Deletion of *vib1* leads to a defect in cellulase production in different improved cellulase producer strains, whereas overexpression had no influence on cellulase expression. These results confirm that *vib1* is an important component of the cellulase regulon in *N. crassa* and *T. reesei*, but the precise function of *vib1* in the regulatory network of *T. reesei* needs to be further examined ([39], this study). However, since straightforward overexpression did not increase the production, a more detailed understanding of the regulatory mechanisms would be required to make this transcription factor interesting for industrial applications by gene engineering strain improvement. Our study demonstrates the importance of studying cellulase-negative strains complementary to high-producing strains for the understanding of the cellulase gene regulation process. It is, to our knowledge, the first report of a distal chromosomal rearrangement in a biotechnology-relevant strain affecting an otherwise intact promoter-gene system, thus provoking a dramatic phenotype. These phenomena should not be overlooked during genome-wide analysis of strains, which often focus on point mutations and InDels.

## Methods

### Fungal strains, culture conditions, and measurement of cellulase formation

*Trichoderma reesei* wild-type strain QM6a (ATCC 13631), the early cellulase producing mutant *T. reesei* QM9414 (ATCC 26921), the cellulase-negative mutant QM9978 (obtained originally from Dr. K.O’ Donnell, U.S. Department of Agriculture, Peoria, IL), and the hypercellulosic strain Rut-C30 (ATCC 56765) were used throughout this study and kept on potato dextrose agar (Sigma, St. Louis, MO) at 30 °C (Additional file 2: Table S1). *T. reesei* was grown in liquid culture in 250-mL Erlenmeyer flasks on a rotary shaker (250 rpm) at 30 °C in 50 mL minimal medium as described [60] using frozen spores as inoculum. Carbon sources used are specified in the legends to the respective figures, and were used as 1% (w/v) in batch cultures. To assess the cellulase formation on different carbon sources using a colorimetric assay based on AZCL-HE-cellulose, strains were grown on minimal medium plates supplemented with 5 g/L of the respective carbon source in addition to AZCL-HE-cellulose (Megazyme International, Ireland) to a concentration of 1 g/L. Determination of protein content in culture supernatants was assessed by the modified Lowry protein assay as described using three biological and at least two technical replicates [61]. Global cellulase activity was measured using the IUPAC standard Filter Paper Assay [62], after a 15-fold miniaturization similar to [63] which allowed working in 2-mL Eppendorf tubes. In order to surround the desired 4% conversion yield, four enzyme dilutions were tested for each sample. This miniaturized protocol was validated by comparison with the standard protocol. Statistical analysis of the results was performed with the Prism GraphPad software (https://www.graphpad.com/scientific-software/prism/).

For transcriptomic studies 50 mL of minimal medium (5 g/L (NH_4_)_2_SO_4_, 5 g/L KH_2_PO_4_, 11.7 g/L trisodium citrate, 0.6 g/L MgSO_4_, 0.6 g CaCl_2_, 20 g/L glucose) were prepared in a 250-mL Erlenmeyer flask. The medium was supplemented with the following trace elements: 0.5 mL/L FeSO4·7H_2_O, 2 mL/L CoCl_2_, 0.16 mL/L MnSO_4_, and 0.14 mL/L ZnSO_4_. Prior to sterilization, the pH of the medium was adjusted to 5.4. Strains QM6a and QM9978 were inoculated in duplicate with approx 10^6^ conidia/mL. Cultivation was carried out at 30 °C at 150 rpm. When the glucose was close to depletion (<3 g/L), 5 mL of a lactose solution was added (25 g/L lactose, 6 g/L (NH_4_)_2_SO_4_, 5 g/L trisodium citrate). Glucose concentration was assessed by enzymatic reaction using an analox glucose analyzer GM10 (Imlab). Extracellular protein concentration was measured against a BSA standard (0–1.5 g/L range with second-order regression) using the Quick Start Bradford Protein Assay kit (Bio-Rad).

To monitor gene expression levels via real-time qPCR, the replacement technique described by Sternberg and Mandels [64] was used: mycelia, grown for 24 h on 1% (w/v) glycerol as the carbon source under otherwise similar conditions as described above, were cautiously filtered through Miracloth (Calbiochem, EMD Biosciences, La Jolla, CA), washed with tap water, and resuspended in minimal medium supplemented with the respective carbon source at 1% (w/v) and the incubation continued for 5, 16, and 24 h. To guarantee equal treatment of the cultures, they were done in parallel and results of three technical replicates were obtained.

*Escherichia coli* NEB 10-beta competent cells (New England Biolabs, France) were used for the propagation of vector molecules and DNA manipulations.

### Vector construction for generation of gene deletion and overexpression mutants

Deletion cassettes consisting of 1–1.5 kb fragments of the gene specific flanking regions interrupted by the hygromycin resistance cassette were assembled by yeast recombinational cloning [50, 65]. Oligonucleotides (10 mM) 5F + 3R and 3F + 3R were used for amplification of the individual flanking regions from genomic *T. reesei* DNA using Phusion polymerase (Thermo Fisher Scientific). Approximately 19 bp that overlap with the pRS426 (URA +) yeast shuttle vector or the *hph* gene were introduced by PCR at each flanking end to allow homologous recombination. The *hph* marker cassette was amplified from pLHhph1 with oligonucleotides hphF and hphR [65]. A PCR touchdown program ranging from 62 to 58 °C for annealing was used for amplification. Oligonucleotide sequences are shown in Additional file 3: Table S2. Yeast transformation was performed as described [66] using the lithium chloride/polyethylene glycol procedure. Yeast strain WW-YH10 (ATCC 208405) was transformed with both flanking regions, the *hph* marker cassette and an* EcoR*I/*Xba*I-digested plasmid pRS426 [67]. Transformants were selected on synthetic complete dropout medium (SC-URA with uracil dropped out). Following total DNA isolation from liquid SC-URA medium, plasmids were introduced into chemically competent NEB 10-beta *E. coli* cells (New England Biolabs) [68]. The outside primers 5F + 3R were used to synthesize the complete deletion cassette from plasmids isolated from *S. cerevisiae*.

For construction of overexpressing strains, the coding region of *vib1* was amplified using oligonucleotides VIB1 for and VIB1 rev and placed under the control of the strong constitutive *gpd* promoter (oligonucleotides promGPD for and PromGPD rev) followed by the *gpd* terminator (termGPD for and termGPD rev) and the *hph* resistance cassette (hph for and hph rev, see Additional file 3: Table S2) all inserted into the *Xba*I/*EcoR*I-linearized plasmid pUC19 yielding plasmid pUC_vib1_OE. Oligonucleotides K7 VIB1 for and K7 VIB1 rev were used to amplify the complete overexpression cassette from pUC_vib1_OE.

For reintroduction of *vib1* under the control of the native promoter into the strain QM9978, the coding region plus approximately 850 bp of the promoter region (after the translocation breakpoint) together with 500 bp of the terminator region were amplified using oligonucleotides VIB1_F and VIB1_R (Additional file 3: Table S2) and introduced in the *Sac*I/*BamH*I-linearized plasmid pLHhph1 using the Gibson Assembly Cloning Kit (New England Biolabs) [65]. Five micrograms of the circular plasmid were used for protoplast transformation of *T. reesei*.

### Transformation of *T. reesei* and analysis of transformants

*Trichoderma reesei* QM9414, QM9978, and Rut-C30 were used for the generation of *vib1* deletion and overexpression strains. Protoplast preparation and transformation were performed as previously described [69]. The deletion cassettes were purified after PCR (QIAquick PCR Purification kit, QIAGEN) and concentrations were determined (Nanodrop Spectrophotometer, Peqlab). After transformation protoplasts were stabilized and regenerated on selection medium supplemented with 100 mg/mL hygromycin B. For sporulation, transformants were transferred to 12-well plates and purified by plating conidiospores onto plates with 0.1% Triton X-100 as colony restrictor. Single colonies were transferred to selective medium and screened for correct integration of the deletion or overexpression cassettes by PCR analysis (primer abbreviated with “ch” in Additional file 3: Table S2). Insertion of the *gpd*-*vib1* overexpression cassette was verified with primer pairs TR46-verif 5′vib1 for + TR47-verif 5′vib1 rev, TR48-verif 3′ vib1 for + TR49-verif 3′vib1 rev, and TR50-verif hph vib1 for + TR51-verif hph vib1 rev amplifying the 5′or 3′ region and the *hph* marker gene, respectively. Three or four independent clones were isolated for each strain construction and analyzed as biological replicates.

### Gene expression analysis by real-time qPCR

DNase treated (DNase I, RNase free; Fermentas) RNA (5 mg) was reverse transcribed with the RevertAidTM First Strand cDNA Kit (Fermentas, Germany) according to the manufacturer’s protocol using a combination (1:1) of the provided oligo-dT and random hexamer primers. All assays were carried out in 96-well plates, which were covered with optical tape, as previously described [70]. Primers, amplification efficiency, and R-square values are given in Additional file 4: Table S3. The amplification efficiency was determined using triplicate reactions from a dilution series of cDNA, and then calculated from the given slopes in the IQ5 Optical system Software v2.0. Expression ratios were calculated using the REST 2009 Software (Quiagen, Germany). All samples were analyzed in two independent experiments with three replicates in each run.

### RNA sample preparation

At each time point, 5 mL culture medium containing mycelia was filtrated on 1.2-µL GF/C glass microfiber membranes and flash frozen in liquid nitrogen. Frozen mycelia were disrupted and homogenized in Lysing Matrix C (Fast RNA Pro Red Kit, MP Bio) in the presence of 700 µL Lysis buffer RLT (RNeasy Mini Kit, Qiagen) complemented with 7 µL of β-mercaptoethanol on a rotor–stator homogenizer (FastPrep, MP Bio, Santa Ana, CA, USA). After centrifugation, the lysate was transferred on a Qiashredder spin column (Qiagen) and centrifuged for 2 min to eliminate all cell debris. The flow-through was then mixed with 0.5 volumes of pure ethanol and transferred to an RNeasy spin column. All further steps, including an on-column DNAse digestion, were realized following the manufacturer’s instructions. RNA quantification was performed with Qubit 2.0 (Life Technologies, Carlsbad, CA, USA). The quantity and quality of the total RNA was determined using a NanoDrop ND-1000 spectrophotometer (Nanodrop Technologies, Wilmington, USA) and by electrophoresis on 1% agarose gel. An additional quality control was performed on the Bioanalyzer 2100 system (Agilent, Santa Clara, USA).

### RNA-seq library preparation and analysis

Library preparation and Illumina sequencing were performed at the Ecole normale superieure genomic platform (Paris, France). Messenger (polyA+) RNAs were purified from 1 µg of total RNA using oligo(dT) primer. Libraries were prepared using the strand non-specific RNA-Seq library preparation TruSeq RNA Sample Prep Kits v1 (Illumina). Libraries were multiplexed by four on one single flowcell lane and subjected to 50-bp paired-end read sequencing on a HiSeq 1500 device. A mean of 52 ± 10 million passing Illumina quality filter reads was obtained for each of all the samples. We performed RNA-seq analysis using the Eoulsan pipeline [71]. Only read 1 was considered for the further analyses. Before mapping, poly N read tails were trimmed, reads ≤11 bases were removed, and reads with quality mean ≤12 were discarded. Reads were then aligned against the *T. reesei genome* (version 2 from the JGI website) using the Bowtie mapper (version 0.12.7) [72]. Alignments from reads matching more than once on the reference genome were removed using Java version of samtools [73]. To compute gene expression, the *T. reesei* genome annotation from JGI (version 2) was used. All overlapping regions between alignments and referenced exons were counted. The sample counts were normalized using DESeq 1.8.3 [74]. Statistical treatments and differential analyses were also performed using DESeq 1.8.3. A threshold of two for the log2 ratios with an adjusted *p* value <0.001 was used to identify genes differentially expressed (DE). Gene functions were identified by the aid of a manually annotated *T. reesei* genome database proprietary to IFPEN.

The RNA-Seq gene expression data and raw fastq files are available at the GEO repository (http://www.ncbi.nlm.nih.gov.gate1.inist.fr/geo/) under Accession Number GSE89199.

### Illumina genome sequencing of *T. reesei* QM9978

Chromosomal DNA from *T. reesei* QM9978 was prepared as previously described [14]. Fragment libraries were prepared according to the TruSeq^®^ DNA Sample Preparation Guide from Illumina (http://www.illumina.com). These libraries were then loaded onto the cluster generation station for single-molecule bridge amplification using the Standard Cluster Generation kit from Illumina. The slide with amplified clusters was then subjected to sequencing on the Illumina Genome Analyser I (GAI) for single reads using the 36 cycle Sequencing Kit version 1 from Illumina.

The whole genome sequence of *T. reesei* QM9978 will be deposited at the Sequence Read Archive (SRA; http://www.ncbi.nlm.nih.gov/sra).

### Sequence alignment and analyses

Illumina short reads from QM9978 were mapped onto the *T. reesei* genome (http://genome.jgi-psf.org/Trire2/), using the Maq 0.6.6 software solution [75]. Mapping was done with two maximum mismatches. InDels and SNVs were identified using Maq 0.6.6. Homozygous mutations were selected and filtered on genomic context (complexity  =  1, uniqueness  > 15.8, GC percentage between 0.31 and 0.74). Each mutation was manually checked using the Integrative Genome Viewer and compared to the sequence of the Rut lineage to remove the false mutations coming from initial QM6a error sequencing. Large structural variations were searched using the BreakDancer software and filtered on the reads number covering the genomic variations [76].

We evaluated the location of SNVs and deletions according to gene annotations using the “filtered models” from the JGI website. From this annotation, we calculated the position of intron, promoter (using an 800 bp upstream region), and terminator (using a 200-bp downstream region).

Functions of genes were predicted by similarity to orthologous genes when found in other fungal taxa using the FUNGIpath database (http://embg.igmors.u-psud.fr/fungipath/). If no orthologous gene function could be identified but a gene with a homology e-value superior to e-10 was identified, the product of this gene was annotated as “similar to.” Conserved domains were identified either by interproscan (http://www.ebi.ac.uk/interpro/search/sequence-search) or by NCBI BLASTp search (https://blast.ncbi.nlm.nih.gov/Blast.cgi).

To validate the translocation between chromosomes V and VII, we amplified the region around genes encoded by the protein IDs 80028 and 54675 by PCR using oligonucleotides up and downstream of the coding region (Primer 54675_MutaC_F + 54675_MutaC_R and 80028_MutaC_F + 80028_MutaC_R Fig. 3b; Additional file 3: Table S2). PCR amplicons were sequenced with adequate primers using Sanger cycle sequencing/capillary electrophoresis (Microsynth AG, Austria).

## Additional files

**Additional file 1: Figure S1.** Growth analysis of *vib1* mutant strains on AZCL-HE-Cellulose. The images correspond to Fig. 4 showing the enzyme production by the strains on AZCL-HE-Cellulose supplemented with 10 g/L lactose or glucose. Comparison of colony size to cellulose degradation shows that strains QM9978 and QM9414 *-vib1* impaired in cellulase production are still able to grow on lactose. Pictures were taken after 4 days of incubation. For each strain two biological replicates (individual clones) were assayed and one is shown representatively.

**Additional file 2: Table S1.** Strains used throughout the study.

**Additional file 3: Table S2.** Oligonucleotides used in this study.

**Additional file 4: Table S3.** Oligonucleotides used for quantitative real-time qPCR analysis.

## Abbreviations

ATCC: American type culture collection
BLAST: basic local alignment search tool
CBH1: cellobiohydrolase 1
CBH2: cellobiohydrolase 2
CRE: carbon catabolite repressor protein
DE: differentially expressed
GH: glycoside hydrolase
HPH: hygromycin B phosphotransferase
Indels: insertions and deletions
JGI: Joint Genome Institute
PDA: potato dextrose agar
QM: Quartermaster (strain collection)
RUT: Rutgers (mutagenesis program)
SNP: short nucleotide polymorphism
SNV: short nucleotide variants
Trire: *Trichoderma reesei*
US: United States
XYR1: xylanase regulator 1
HPH: hygromycin B phosphotransferase
