# Supplementary figures and images for: Genome sequencing and transcriptome analysis of Trichoderma reesei QM9978 strain reveals a distal chromosome translocation to be responsible for loss of vib1 expression and loss of cellulase induction
Source: Biotechnol Biofuels. 2017 Sep 7;10:209. doi: 10.1186/s13068-017-0897-7 (PMC5588705; doi:10.1186/s13068-017-0897-7)

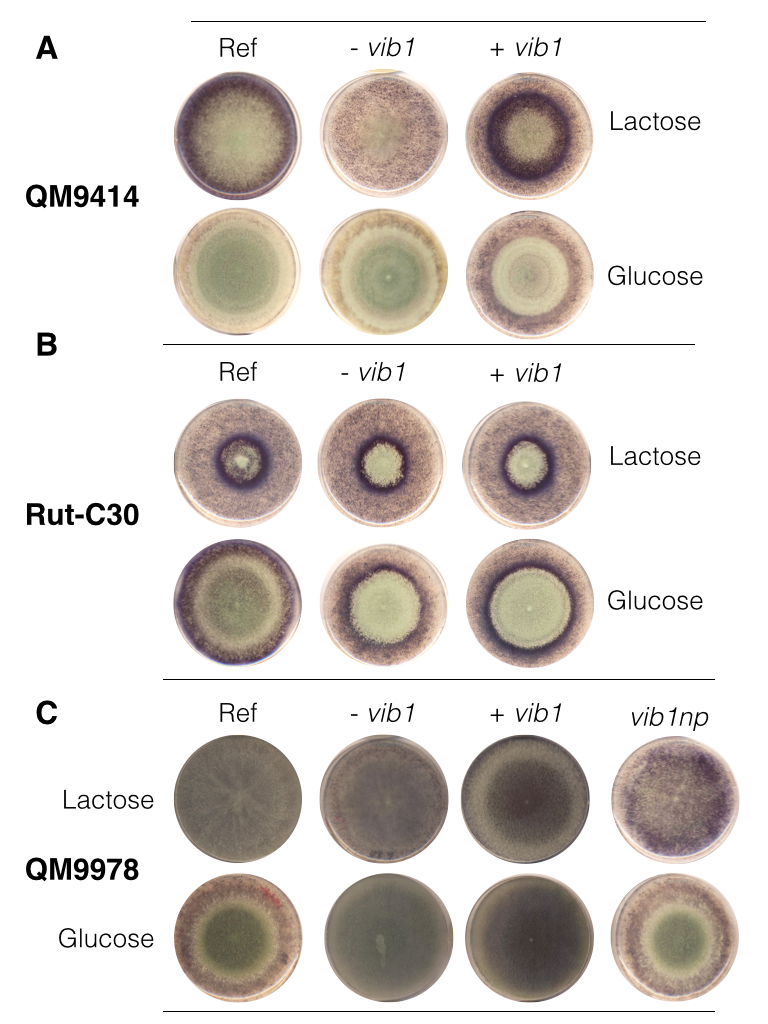

Supplement: Supplementary file 1 — Additional file 1: Figure S1. Growth analysis of vib1 mutant strains on AZCL-HE-Cellulose. The images correspond to Fig. 4 showing the enzyme production by the strains on AZCL-HE-Cellulose supplemented with 10 g/L lactose or glucose. Comparison of colony size to cellulose degradation shows that strains QM9978 and QM9414 -vib1 impaired in cellulase production are still able to grow on lactose. Pictures were taken after 4 days of incubation. For each strain two biological replicates (individual clones) were assayed and one is shown representatively. [file 13068_2017_897_MOESM1_ESM.tiff]
