# Supplementary material for: Genome sequencing and transcriptome analysis of Trichoderma reesei QM9978 strain reveals a distal chromosome translocation to be responsible for loss of vib1 expression and loss of cellulase induction
Source: Biotechnol Biofuels. 2017 Sep 7;10:209. doi: 10.1186/s13068-017-0897-7 (PMC5588705; doi:10.1186/s13068-017-0897-7)
Supplement: Supplementary file 2 — Additional file 2: Table S1. Strains used throughout the study. [file 13068_2017_897_MOESM2_ESM.docx]

**Table S1.** Strains used throughout the study.

| **Strain** | **Genotype** | **Reference** |
| --- | --- | --- |
| QM6a  QM9414 | - | Mandels *et al.,* 1971 [[48](#_ENREF_48)]  Mandels *et al.,* 1971 [[48](#_ENREF_48)] |
| QM9414 *Δvib1* | *Δvib1::hph* | this study |
| QM9414 *vib1OE* | P*gpd::vib1::*T*gpd::hph* | this study |
| QM9978 | - | Torigoi *et al.,* 1996 [[7](#_ENREF_7)] |
| QM9978 *Δvib1* | *Δvib1::hph* | this study |
| QM9978 *vib1OE* | P*gpd::vib1::*T*gpd::hph* | this study |
| QM9978 *vib1np* | P*vib1::vib1::*T*vib1::hph* | this study |
| Rut-C30 | - | Eveleigh & Montenecourt 1977 [[6](#_ENREF_6)] |
| Rut-C30 *Δvib1* | *Δvib1::hph* | this study |
| Rut-C30 *vib1OE* | P*gpd::vib1::*T*gpd::hph* | this study |
