# Supplementary material for: Genome sequencing and transcriptome analysis of Trichoderma reesei QM9978 strain reveals a distal chromosome translocation to be responsible for loss of vib1 expression and loss of cellulase induction
Source: Biotechnol Biofuels. 2017 Sep 7;10:209. doi: 10.1186/s13068-017-0897-7 (PMC5588705; doi:10.1186/s13068-017-0897-7)
Supplement: Supplementary file 3 — Additional file 3: Table S2. Oligonucleotides used in this study. [file 13068_2017_897_MOESM3_ESM.docx]

**Table S2.** Oligonucleotides used in this study.

| **Name** | **Sequence (5’ – 3’)** | **Usage** |
| --- | --- | --- |
| VIB1_F | CCAGTGAATTCGAGCTTATTATGCCACCGGATGTC | *vib1* gene |
| VIB1_R | TGTAAGGTAGCTCTCGGCCTAGGTAGTCACTCATC | deletion |
| hphF | GGATCCGAGAGCTACCTTAC | constructs |
| hphR | CTCGAGGGTACTATGGCTTA |  |
| deltaVIB1_5F | GTAACGCCAGGGTTTTCCCAGTCACGACGGACTCTGTTCTTACGCCTTG |  |
| deltaVIB1_5R | CATATTGATGTAAGGTAGCTCTCGGATCCAAATAGCCCAGCAACTCGC |  |
| deltaVIB1_3F | TATTCCATCTAAGCCATAGTACCCTCGAGTTGATACTGCGGCGTCTGT |  |
| deltaVIB1_3R | GCGGATAACAATTTCACACAGGAAACAGCCTCAGTACCTACCGCGTC |  |
| Vib1chr | GGATACCAACGTGTGAACCT |  |
| VIB1_rev_ch | GGCGTAGAATCGTCAACTG |  |
| deltaVIB1_ch | CATACACGCACACATCCTC |  |
| deltaVIB1_9978FW | GTAACGCCAGGGTTTTCCCAGTCACGACGGGTCTCACTTCTCTCGGCG |  |
| deltaVIB1_9978RV | CATATTGATGTAAGGTAGCTCTCGGATCCGGTCACTGGATATGCTGTT |  |
| promGPD For | GCCTGCAGGTCGACTGACGCAGAAGAAGGAAATC |  |
| PromGPD rev | TCGGTCATTTTGTATCTGCGAATTGAGC |  |
| VIB1 for | GCCTGCAGGTCGACTGACGCAGAAGAAGGAAATC | *vib1* |
| VIB1 rev | TCGGTCATTTTGTATCTGCGAATTGAGC | overexpression |
| termGPD for | CAGATACAAAATGACCGACCTCAGAGGAG |  |
| termGPD rev | CACAGCACTTAATTACCATGCCAGGAGTAG |  |
| hph for | TGGTAATTAAGTGCTGTGTTCCTCAGAATG |  |
| hph rev | AAACGACGGCCAGTGGGCAGTGCTAGTGTGTGTAC |  |
| K7 VIB1 for | GACGCAGAAGAAGGAAATCGC |  |
| K7 VIB1 rev | GGTACACACACTAGCACTGCC |  |
| TR46-verif 5'vib1 for | GTCAGAAACGACCAAGCTAAG | verification of |
| TR47-verif 5'vib1 rev | TGATCCACGATGGTGTGC | construct |
| TR48-verif 3' vib1 for | CCGAGTACCGATACGGATCG | insertion |
| TR49-verif 3'vib1 rev | GACGCCTGAGAGGTCCTA |  |
| TR50verif hph vib1 for | CCTTCTTAGAGAGCTCTCGG |  |
| TR51-verif hph vib1 rev | CGGGTTTACCTCTTCCAGAT |  |
| 80028_MutaC_F | TTGGAGCTGGAGTCGATGATTG | verification of |
| 80028_MutaC_R | GGAAGATGAACGTGATTGGTCG | translocation |
| 54675_MutaC_F | CGCCTCTTTGCCTTCTTGACTG |  |
| 54675_MutaC_R | GGCGCAGTGAACTTGTGGTG |  |
