# Supplementary material for: Genome sequencing and transcriptome analysis of Trichoderma reesei QM9978 strain reveals a distal chromosome translocation to be responsible for loss of vib1 expression and loss of cellulase induction
Source: Biotechnol Biofuels. 2017 Sep 7;10:209. doi: 10.1186/s13068-017-0897-7 (PMC5588705; doi:10.1186/s13068-017-0897-7)
Supplement: Supplementary file 4 — Additional file 4: Table S3. Oligonucleotides used for quantitative real-time qPCR analysis. [file 13068_2017_897_MOESM4_ESM.docx]

**Table S3.** Oligonucleotides used for quantitative real-time qPCR analysis.

| **Gene ID** | **Oligonucleotide** | **Sequence (5’ - 3’)** | **Amplification**  **Efficiency [%]** | **R^2^ value** |
| --- | --- | --- | --- | --- |
| 123902/*tef1* | tef1 qPCR fw | CCACATTGCCTGCAAGTTCGC | 91 | 0.998 |
|  | tef1 qPCR rv | GTCGGTGAAAGCCTCAACGCAC |  |  |
| 61470/*sar1* | sar1fw | TGGATCGTCAACTGGTTCTACGA | 95 | 0.998 |
|  | sar1rev | GCATGTGTAGCAACGTGGTCTTT |  |  |
| 123989/*cbh1* | cbh1 qPCR fw | CCGAGCTTGGTAGTTACTCTG | 101 | 0.999 |
|  | cbh1 qPCR rv | GGTAGCCTTCTTGAACTGAGT |  |  |
| 122208/*xyr1* | qPCR_xyr1_for | CCATCAACCTTCTAGACGAC | 105 | 0.997 |
|  | qPCR_xyr1_rev | AACCCTGCAGGAGATAGAC |  |  |
| 120117/*cre1* | cre1 qPCR fw | ACAGTCTGCCGTGGATTT | 98 | 0.998 |
|  | cre1 qPCR rv | TCTCGGTCGACTGTGTTC |  |  |
